# The Pathology and Treatment of Pulmonary Tuberculosis

**Published:** 1854-09

**Authors:** 


Art. V.—*The Pathology and Treatment of Pulmonary Tuberculosis;* and of the local medication of Pharyngeal and Laryngeal diseases frequently mistaken for, or associated with, Phthisis. By John Hughes Bennett, M. D., F. R. S. E., Professor of the Institutes of Medicine and of Clinical Medicine in the University of Edinburgh; Fellow and Censor of the Royal College of Physicians, Edinburgh; Member of the American Philosophical Society, and of various Medical Societies in Edinburgh, Paris, Vienna, Berlin, Stockholm, Copenhagen, &c., &c. Philadelphia: Blanchard & Lea. 1854.

---

In his preface to this work Dr. Bennett remarks: "In the years 1839–40, the author found a remedy administered in the hospitals of Germany in cases of Pulmonary Tuberculosis, which had never been employed in his own country for that disease, although it had been successfully used there in rheumatism. This was Cod-liver Oil. He could not fail to be struck with the marked benefits which resulted from its employment in patients, who, had they been treated in British hospitals, would certainly have died. In 1841, therefore, he published a Monograph, giving an account of what was then known concerning that substance, and recommended it to his countrymen, from theoretical and practical considerations, as a valuable remedy in Phthisis Pulmonalis.

"For five years (1843–1848), the author held the position of Pathologist to the Royal Infirmary of this city, during which period he performed and recorded the results of upwards of two thousand *post-mortem* examinations. Gradually, one great fact became impressed upon his mind, viz: that all organic diseases occasionally presented a tendency to spontaneous cure. He was repeatedly meeting with instances where, although death was occasioned by disease in one organ, there were others which presented traces of previously existing lesions which in some way had healed. In no organs were such appearances more common than in the lungs, and of no disease was evidence of a spontaneous cure more frequent than of Pulmonary Tuberculosis.

"Although it was generally considered by the profession that no remedy and no plan of treatment yet proposed could be depended on in cases of consumption, it was obvious to the author that if the process employed by nature could be discovered, and then imitated by art, we might ultimately arrive at the true principle of cure. Whenever, therefore, decided and unmistakable evidence of a spontaneous cure came before him, he carefully studied the circumstances which preceded it; and connecting these with the numerous observations which were simultaneously going on, as to the good effects of Cod liver Oil, he was gradually led to the rules of treatment which are developed in the following pages. These he has tested on a large scale in hospital and private practice. They have also been extensively tried by others who have followed his suggestions, so that he can now confidently recommend them to his professional brethren."

The multiplication of works on *tuberculosis*, within a few years, is gratifying evidence that the profession is beginning to feel confidence in the curability of consumption. Despair has given place to rational hope and scientific effort. Statistics bear testimony to the value of our altered mode of medication. The author of this work was one of the first English practitioners to adopt the practice which has been attended with such encouraging results. He contends that tubercular diseases will heal of themselves if the faulty nutrition of the system can be removed; and that, with this object, our efforts should be directed to the digestive rather than to the respiratory system. The kind of abnormal nutrition which exists is dependent, he thinks, on increased assimilation of the albuminous, and diminished assimilation of the fatty portions of the food, and hence the plan of treatment is to cause the reception of the deficient elements.

We pass over all that Dr. BENNETT says on the pathology and nature of tubercle, to notice some more practical subjects. After discussing at some length the *arrestment* of pulmonary tuberculosis, he concludes with these remarks:—

"What has been now stated, must, I think, show that the arrestment of tubercular ulceration may take place in three ways: 1st. By the gradual transformation of the exudation into cretaceous and calcareous concretions. 2nd. By expectoration and absorption of the exudation, the collapse of the ulcerated walls, and formation of a cicatrix. 3rd. By the ulcerated walls becoming covered with a smooth membrane, remaining open, and constituting chronic cavities, which have occasionally been mistaken for dilated bronchi. It should not be overlooked that one or all of these modes of arrestment may be detected in the same lung, and that great diversities of appearance in the pulmonary texture may be thus occasioned. Moreover, the occurrence of black carbonaceous deposits is very apt to take place in the neighborhood of the cretaceous concretions and cicatrices, thus communicating increased density to the texture. This density and contraction in the pulmonary tissue also is, as Dr. Gardiner has shown, a cause of pulmonary emphysema in the air vesicles, a circumstance which has attracted the attention of many pathologists, and has even been considered to be essentially connected with the spontaneous cure of pulmonary caverns (Ramadge). This occasional result, however, is much to be dreaded, as it causes the dyspnœa not unfrequently observed to follow the removal of pulmonary tubercle. But the frequency with which all these various lesions are discovered, and their connection with the spontaneous cure of pulmonary tuberculosis, points out how much more commonly this lesion is arrested, than for a long period has been generally believed by the profession.

"From the preceding facts and observations, therefore, we are warranted in drawing the conclusion, that if during the advance of phthisis pulmonalis, those means can be discovered which check further tubercular exudation, and keep up the strength and nutritive processes of the economy, such tubercular exudations as have occurred will be rendered abortive, and that even large ulcerations will heal up and cicatrize. The important point practically is to ascertain what these means are, and how they may be put into operation."

His principles of *treatment* are thus stated:—

"From a study of the symptoms, causes, morbid anatomy, and histology of pulmonary tuberculosis, we have been led to the conclusion that it is a disease of the primary digestion, causing: 1st. Impoverishment of the blood. 2nd. Exudations into the lung, which present the characters of tubercular exudation. 3rd. Owing to the successive formation and softening of these, and the ulcerations which follow in the pulmonary or other tissues, the destructive results which distinguish them. It has also been shown that circumstances which remove the mal-assimilation of food frequently check the tendency to repeated tubercular exudations, while those which previously existed become abortive, and that occasionally extensive excavations in the pulmonary tissue may, owing to like circumstances, heal up and cicatrize.

"But although the fact of the recovery from phthisis pulmonalis, even in its most advanced stage, can no longer be denied, it has been argued that this is entirely owing to the operations of nature, and that the physician can lay little claim to the result. Andral, who early admitted the occasional cicatiization of caverns, states this in the following words: "No fact," he says, "demonstrates that phthisis has been ever cured, for it is not art which operates in the cicatrization of caverns; it can only favor this, at most, by not opposing the operation of nature. For ages, remedies have been sought either to combat the disposition to tubercles or to destroy them when formed, and thus innumerable specifics have been employed and abandoned in turn, and chosen from every class of medicaments." But if it be true, according to Hoffman, that '*Medicus naturæ minister non magister est*,' it follows that, by carefully observing the operations of nature, learniug her method of cure, imitating it as closely as possible, avoiding what she points out to be injurious, and furnishing what she evidently requires, we may at length arrive at rational indications of treatment. In short, a correct interpretation of the processes gone through during a spontaneous arrestment of the disease, must be the foundation for those principles which should regulate the interference of art.

"Now, a careful examination of phthisical cases will, I think, show that the great obstacles the practitioner has to contend with are the dyspeptic symptoms, which render all his efforts at nourishing the patient in the ordinary way useless. Such individuals have a most capricious appetite, frequently loathe all kinds of animal food; and it will be found that, even when they say that the appetite is good, and that they live well, the diet actually consumed is either deficient in quantity or in quality. Nothing, again, is more common in the progress of such cases than the temporary improvements which follow a change of diet, of locality, or of temperature. How frequently do poor patients, on coming into an hospital, get better merely from enjoying rest and the regular diet of the institution. How often, after a short journey, or on reaching what has been considered a favorable locality, are the friends of consumptive patients in the higher classes rendered happy by the temporary marked improvement which takes place. I consider that such amendments will always be found commensurate to the stimulus given to the nutritive processes of the economy.

"From the foregoing considerations, it follows that the cure of pulmonary tuberculosis by art, will be proportionate to our power, 1. Of improving the faulty nutrition which is the cause of the exudation assuming a tubercular character. 2. Of favoring absorption of the exudation already poured out; and 3. Of preventing the recurrence of fresh exudations by careful attention to hygienic regulations. I propose making some observations on each of these heads, as preliminary to the special treatment of individual cases."

In regard to cod liver oil he says:—

"Since I introduced cod-liver oil to the notice of the profession in this country, as a remedy for phthisis, in 1841, I have continually prescribed it in hospital, dispensary, and private practice. I need not perhaps say that I have given it in a very large number of cases, and have observed its effects in all stages of the disease, and under almost every circumstance of age, sex, and condition. I have had the most extensive opportunities of examining the bodies of those who have died after taking it in considerable quantities, and am still observing the cases of many persons who may be said to have owed their lives to its employment. Further I have carefully watched the progress it has made in the good opinion of the professional public, and perused all that has been published regarding it in the literature of this and other countries. It were certainly easy for me, therefore, to write at great length on this subject; but it appears to me unnecessary to dwell on the utility of a remedy which the experience of the last twelve years in Scotland, and twenty years in Germany, has rendered so obvious. It need only be said with regard to cod-liver oil, that, although at first many eminent practitioners have felt more or less skeptical as to its value, and have therefore delayed giving it, I have not heard of one who, after employing it in a few cases, has retained any doubt as to its importance in scrofulous and consumptive cases.

"The general opinion of the profession, with regard to cod-liver oil, may be said to have fully confirmed what I formerly stated concerning it, in the following words, viz: "That no remedy has so rapidly restored the exhausted powers of the patient, improved the nutritive functions generally, stopped or diminished the emaciation, checked the perspiration, quieted the cough and expectoration, and produced a most favorable influence on the local disease. Many individuals presenting the emaciation, profuse sweats, constant cough and expectoration, as most prominent symptoms, with a degree of weakness that prevents their standing alone, after a few weeks use of it, are enabled to get up with ease and walk about, with a visible improvement in their general health, and an increased amount of flesh. The physical signs of the disease may continue unaffected for some time, but if the treatment be continued, the moist gurgling rattles are exchanged for dry blowing sounds, which become more and more persistent, pectoriloquy is merged into bronchophony, the respiration is easier and a check is evidently given to the ulcerative process, and the formation of purulent matter in the air passages."

"The following is a summary of my views regarding cod-liver oil, as a remedy for pulmonary tuberculosis:—

"1. Cod-liver oil is, as M. Taufflieb pointed out, an *analeptic* and is indicated in all cases of deranged nutrition dependent on want of assimilation of fatty matter.

"2. It is readily digestible under circumstances where no other kind of animal food can be taken in sufficient quantity to furnish the tissues with a proper amount of fatty material.

"3. It operates by combining with the excess of albuminous constituents of the chyme, and forming in the villi and terminal lacteals those elementary molecules, of which the chyle is originally composed.

"4. Its effects in phthisis are, to nourish the body, which increases in bulk and in vigor; to check fresh exudations of tubercular matter; and to diminish the cough, expectoration, and perspiration.

"5. The common dose for an adult is a table-spoonful three times a day, which may often be increased to four, or even six, with advantage. When the stomach is irritable, however, the dose to commence with should be a tea or dessert spoonful.

"6. The kind of oil is of little importance therapeutically. The pure kinds are most agreeable to the palate; but the brown coarser kinds have long been used with advantage, and may still be employed with confidence whenever cheapness is an object.

"7. I have never observed its employment to induce pneumonia, or fatty disease of the liver or kidney, however long continued, although such complications of phthisis are exceedingly frequent."

Dr. Bennett says he has seen mutton and pork chops do wonders in tuberculosis, and is convinced that other oily articles of food have occasionally been productive of great benefit; but he adds, as the result of much experience, that "no substance hitherto known is so easily tolerated by the stomach, and is of such general application as an analeptic in tubercular diseases, as cod-liver oil."

He adds:—

But even this substance, in a few rare cases, cannot be rétained on the stomach, and efforts have been make to introduce fat into the economy by some other channel, such as by the skin or rectum. Frictions with oil and unguents were largely employed by the ancients, and constituted part of the system of training their athletæ. More recent observations have shown that butchers, cooks, oil-men, tallow-chandlers, tanners, and other individuals who are continually coming in contact with fatty matter, are for the most part particularly robust and well nourished, and are less liable than others to tubercular disease. Dr. Simpson has lately noticed, that the children and young persons employed in wool factories, where large quantities of oil are daily used, are generally exempt from scrofula and from pulmonary consumption. This renders it probable that a certain amount of fat may, by entering the lymphatics of the skin, so affect the chyle as to render it more nutritive; and that, in cases where oil cannot, without great difficulty be tolerated by the stomach, advantage may be derived from introducing it through the integument. Such a practice, indeed, has been recommended by Dr. Baur of Tubingen, who has given several instances where the scrofulous disposition has been removed by rubbing in different kinds of oil. I have adopted this plan in a few cases, but having rarely met with patients who could not be induced to take the oil internally, under proper management, my experience of its effects, administered by the skin, has been very limited. Some time ago, I caused a cod-liver oii ointment, manufactured by the Messrs. Parker, of Leith Walk, to be used externally; but, unfortunately, the constant smell proved to be more disagreeable than the unpleasant taste and eructations. Even the purest vegetable oils, used externally, cause great trouble in applying them, stain the linen and dress, occasion in some, unpleasant sensations of uncleanliness, and, to the poor, are very costly. Still if oil be assimilated, however administered, it must, according to the principles I am contending for, be beneficial; and as the practice of inunction is now being very extensively tried, we shall soon have more exact means of judging of its value."

The remarks of Dr. Bennett on diet are highly judicious, and, in fact, all his practical observations are of so much interest that we regret our inability to make more numerous and more copious extracts. This regret however is diminished by the circumstance that the work, being a small one, and at the same time, one of so much importance, is sure to arrest the attention of practitioners and be universally read.

